# Iatrogenic Dextrose Hyperosmolar Extravasation Injury in Suspected Endogenous Hyperinsulinism

**DOI:** 10.7759/cureus.115223

**Published:** 2026-08-26

**Authors:** Wesley A Kim, Andrew Latef, Sherif Latef, Jericho Oviedo

**Affiliations:** 1 Diagnostic Radiology Department, Rowan-Virtua School of Osteopathic Medicine, Stratford, USA; 2 Intensive Care Unit, Hudson Regional Health Hospital, Secaucus, USA

**Keywords:** bullous skin lesions, central venous access, dextrose extravasation, endogenous hyperinsulinism, extravasation injury, hyperosmolar infusion, hypoglycemic seizure, insulinoma, peripheral intravenous catheter, refractory hypoglycemia

## Abstract

Hyperosmolar dextrose infused through peripheral venous access can extravasate into surrounding soft tissue and produce local injury. We present the case of a 44-year-old woman with a history of seizure disorder and hyperlipidemia who underwent anterior lumbar interbody fusion (ALIF). On the day of admission, she experienced a witnessed generalized seizure on the floor that terminated following administration of intravenous lorazepam. Point-of-care glucose obtained during the event was 39 mg/dL and corrected with intravenous dextrose. She was transferred to the intensive care unit (ICU), where hypoglycemia recurred despite continuous dextrose support. Computed tomography (CT) of the head showed no acute intracranial process, and electroencephalography (EEG) demonstrated diffuse slowing without epileptiform activity. Endocrinology was consulted, and the clinical picture was felt to be most consistent with endogenous hyperinsulinism from a suspected insulinoma. At a serum glucose of 55 mg/dL, insulin was 13 uIU/mL and C-peptide was 4.7 ng/mL, both inappropriately unsuppressed and consistent with endogenous hyperinsulinism. She was maintained on continuous 10% dextrose at 150-200 mL per hour with as-needed 50% dextrose boluses, glucagon, and subcutaneous octreotide 200 mcg every eight hours, with capillary glucose monitoring every two hours.

On hospital day 4, the peripheral catheter site was found to be infiltrated, and the catheter was removed, with the infusion transferred to central access. On hospital day 6, nursing and physician staff identified multiple tense, fluid-filled bullae over the right anterior forearm with associated swelling and erythema at the site of a previously removed peripheral intravenous catheter. The patient reported that forearm tenderness had been present since admission and had progressively worsened. Examination demonstrated moderate asymmetric swelling of the right upper extremity with intact distal pulses and preserved sensation. Range of motion was limited by swelling and a sensation of heaviness rather than by pain. The findings were felt to be most consistent with hyperosmolar dextrose extravasation injury. This case demonstrates that sustained peripheral administration of concentrated dextrose can produce progressive bullous and soft tissue injury and that these changes may evolve insidiously over several days. Early changes may be subtle and difficult to distinguish from routine infusion site irritation. Patients with suspected endogenous hyperinsulinism often require a multiday dextrose bridge while localization and definitive treatment are arranged, which places them at particular risk. Early consideration of central venous access, structured infusion site surveillance, and prompt escalation when local changes are identified are important components of care in patients requiring prolonged high-concentration dextrose therapy.

## Introduction

Extravasation is the unintended escape of infused fluid from a vein into the surrounding soft tissue [1]. When the escaped fluid is hyperosmolar, the injury is driven mainly by osmotic force [2]. Water is pulled out of nearby cells along the newly created gradient, dehydrating them and damaging capillary endothelium [2]. Local perfusion falls, and viable tissue can progress to blistering and frank necrosis [3]. Severity tracks with solute concentration, and experimental work has shown that glucose becomes a vesicant above a threshold osmolarity [3]. Both dextrose solutions used in this patient are hypertonic. A 10% dextrose has a calculated osmolarity of 505 mOsm/L, while a 50% dextrose is roughly fivefold more concentrated [4]. Because of this risk, product labeling and nutrition standards advise a central vein for solutions at or above 900 mOsm/L, as peripheral delivery above that threshold favors phlebitis, vein damage, and tissue injury [4,5].

Extravasation from peripheral catheters is common in hospitalized adults. A meta-analysis of 76,977 catheters found a pooled infiltration or extravasation rate of 13.7%, second only to phlebitis among noninfectious complications [6], and a prospective cohort reported infiltration in 16.3% of cannulas [7]. Despite this frequency, dextrose is thinly represented in the extravasation literature, which is dominated by vasopressors, chemotherapy agents, and iodinated contrast media [2]. Dextrose-specific reports are limited to isolated cases of 50% dextrose given for acute hypoglycemia, one describing forearm compartment syndrome after prehospital extravasation and another describing hyaluronidase treatment after infiltration in the emergency department [8,9]. No case series or incidence estimates specific to prolonged dextrose infusion were identified.

Endogenous hyperinsulinism causes hypoglycemia through inappropriate insulin secretion that persists despite low glucose. Insulinoma is the most common cause in adults without diabetes, with an estimated incidence of one to four cases per million per year [10]. Localization, staging, and operative planning take time, and definitive therapy is often not immediate [10]. During that interval, hypoglycemia can be severe and life-threatening, and somatostatin analogues, diazoxide, and glucocorticoids do not always achieve euglycemia [11]. Patients with refractory disease therefore require hospitalization with continuous intravenous dextrose as a bridge until artificial nutrition is established or the tumor is treated [12]. That bridging requirement is where management of hyperinsulinemic hypoglycemia collides with the hazards of hyperosmolar infusion, because sustained high-concentration dextrose must be delivered somewhere and peripheral delivery carries the osmolar risk described above [4,12].

This report addresses that gap. The extravasation literature documents harm from single boluses of 50% dextrose, but not from the repeated vesicant boluses and sustained hypertonic infusion this care demands [8,9]. This case describes hyperosmolar dextrose extravasation injury arising during continuous dextrose therapy for suspected endogenous hyperinsulinism. The aim is to draw attention to a recognizable and preventable complication rather than to claim a wholly novel entity.

## Case presentation

A 44-year-old woman was admitted after undergoing elective anterior lumbar interbody fusion (ALIF) for symptomatic lumbar spondylolisthesis. Her history included a seizure disorder and hyperlipidemia. She was not diabetic and took no insulin or oral hypoglycemic agents at home. She had been prescribed levetiracetam but was documented as noncompliant, and her seizure frequency had increased progressively over the past three years before admission. On hospital day 1, following the ALIF, she had a witnessed generalized seizure on the medical-surgical floor. The seizure stopped after intravenous lorazepam. Point-of-care glucose during the event was 39 mg/dL. The hypoglycemia was corrected with intravenous dextrose. She was transferred to the intensive care unit (ICU) for monitoring.

Hypoglycemia recurred despite dextrose support. On hospital day 2, there was concern for a brief seizure at approximately 1500. No further seizure episodes occurred after that time. The patient tolerated an oral diet throughout the admission and required no enteral feeding. High-carbohydrate foods and juices were provided in addition to the continuous dextrose infusion. Hypoglycemia persisted despite both oral intake and continuous intravenous dextrose. Endocrinology and Neurology were both consulted. Suspicion centered on endogenous hyperinsulinism.

Diagnostic studies were undertaken to identify the cause of the recurrent hypoglycemia and to exclude alternative explanations. Computed tomography (CT) of the head on hospital day 1 showed no acute intracranial process. Electroencephalography (EEG) on hospital day 3 showed diffuse slowing without seizure activity. Magnetic resonance imaging (MRI) of the head demonstrated no pituitary or other structural lesion to account for an endocrine cause of hypoglycemia, and no epileptogenic focus was identified. Transthoracic echocardiography on hospital day 4 was grossly normal. Right upper quadrant ultrasound on hospital day 4, obtained during the evaluation of mildly abnormal liver enzymes, showed gallbladder sludge without stones or signs of cholecystitis. Hemoglobin A1c was 5.18%, and morning cortisol was within normal limits. Thyroid-stimulating hormone was initially low, with a repeat value within normal limits. Ammonia was mildly elevated on two occasions. C-reactive protein on hospital day 5 was elevated and was felt to be reactive following the ALIF. Folate and vitamin B12 were within normal limits, and vitamin D was low. The lipid profile on hospital day 2 was within normal limits. Lactate was normal. At a serum glucose of 55 mg/dL, insulin was 13 uIU/mL (reference range: 2.6-24.9 uIU/mL), and C-peptide was 4.7 ng/mL (reference range: 0.8-3.9 ng/mL). Neither was suppressed at that glucose, a pattern consistent with endogenous hyperinsulinism and raising suspicion for insulinoma. Pancreatic localization studies were pending at the time of reporting. Selected laboratory values across the hospital course are shown in Table 1.

**Table 1:** Selected laboratory values across the hospital course Arrows indicate values above (↑) or below (↓) the reference range. Glucose values were obtained while the patient was receiving a continuous 10% dextrose infusion and do not reflect the untreated glycemic state. Magnesium and phosphorus were not measured on every hospital day. K/uL: thousand per microliter, g/dL: grams per deciliter, mmol/L: millimoles per liter, mg/dL: milligrams per deciliter, U/L: units per liter

Laboratory marker	Reference range	Hospital day 3	Hospital day 4	Hospital day 5	Hospital day 6
White blood cell count	4.0-10.4 K/uL	19.8 K/uL ↑	12.6 K/uL ↑	14.0 K/uL ↑	11.8 K/uL ↑
Hemoglobin	11.5-15.5 g/dL	11.2 g/dL ↓	10.9 g/dL ↓	11.8 g/dL	10.9 g/dL ↓
Hematocrit	36.0%-48.0%	35.8% ↓	35.5% ↓	36.3%	34.3% ↓
Platelet count	140-400 K/uL	172 K/uL	167 K/uL	211 K/uL	189 K/uL
Sodium	137-145 mmol/L	138 mmol/L	137 mmol/L	138 mmol/L	139 mmol/L
Potassium	3.5-5.1 mmol/L	3.6 mmol/L	3.5 mmol/L	3.5 mmol/L	3.3 mmol/L ↓
Bicarbonate	22-30 mmol/L	27 mmol/L	29 mmol/L	32 mmol/L ↑	34 mmol/L ↑
Blood urea nitrogen	7-18 mg/dL	3 mg/dL ↓	3 mg/dL ↓	4 mg/dL ↓	3 mg/dL ↓
Creatinine	0.67-1.17 mg/dL	0.7 mg/dL	0.7 mg/dL	0.7 mg/dL	0.7 mg/dL
Glucose	74-100 mg/dL	131 mg/dL ↑	190 mg/dL ↑	175 mg/dL ↑	169 mg/dL ↑
Aspartate aminotransferase	14-36 U/L	77 U/L ↑	56 U/L ↑	110 U/L ↑	107 U/L ↑
Alanine aminotransferase	9-52 U/L	31 U/L	28 U/L	43 U/L	45 U/L
Albumin	3.5-5.0 g/dL	3.3 g/dL ↓	2.9 g/dL ↓	3.2 g/dL ↓	2.9 g/dL ↓
Magnesium	1.6-2.3 mg/dL	-	-	-	1.6 mg/dL
Phosphorus	2.5-4.5 mg/dL	-	-	2.1 mg/dL ↓	2.5 mg/dL

Several laboratory abnormalities were noted during the hospital course. The leukocytosis downtrended from 19.8 K/uL to 11.8 K/uL without antibiotic therapy, consistent with a postoperative inflammatory response. Transaminases were mildly elevated with an aspartate aminotransferase-predominant pattern. Blood urea nitrogen remained persistently low, consistent with high-volume dextrose administration. A mild metabolic alkalosis and hypoalbuminemia were also present. No additional workup was pursued for these findings, and none altered management.

Glycemic management relied on continuous dextrose infusion. A continuous 10% dextrose infusion was started at 150-200 mL/hr. Four boluses of 50% dextrose were given as needed for hypoglycemia. Two were administered on hospital day 1 at approximately 0600 and 1400. Two were administered on hospital day 2 at approximately 0600 and 1400. Capillary glucose was monitored every 2 hours per Endocrinology. Glucagon was available as needed. Octreotide 200 mcg was given subcutaneously every 8 hours. The continuous 10% dextrose infusion and all four 50% dextrose boluses were administered through a single 20-gauge peripheral intravenous catheter placed in the right antecubital fossa on hospital day 1. Other medications during the ICU course included lacosamide 200 mg intravenously every 12 hours, lorazepam 2 mg intravenously every 6 hours as needed for seizures, oxycodone 5 mg orally every 4 hours as needed for pain score 1-3, oxycodone 10 mg orally every 4 hours as needed for pain score 4-6, cyclobenzaprine 10 mg orally every 8 hours as needed for muscle spasm, acetaminophen 975 mg orally every 8 hours scheduled, ondansetron 4 mg intravenously every 4 hours as needed for nausea, rosuvastatin 10 mg orally at bedtime, enoxaparin 40 mg subcutaneously daily with sequential compression devices, and pantoprazole 40 mg intravenously daily.

On hospital day 4, the right forearm was noted to be erythematous and edematous at the catheter site, without bullae, ulceration, blanching, or induration at that time, and the peripheral catheter was documented as infiltrated. The catheter was removed, giving a total dwell time of three days. Repeated peripheral access failures prompted escalation to central access. A 5-French double-lumen peripherally inserted central catheter (PICC) was placed in the left brachial vein under ultrasound guidance the same day, with the indication documented as continuous 10% dextrose and frequent laboratory draws. Position was confirmed by chest radiograph. The 10% dextrose infusion was continued through this catheter and later reduced to a maintenance rate of 75 mL/hr.

Vital signs on hospital day 6 included a temperature of 98.1 F, blood pressure of 117/62 mmHg, heart rate of 85 bpm, oxygen saturation of 98% on room air, and respiratory rate of 20/min. Across the day, temperature ranged from 98.1°F to 100.4°F, heart rate from 85 bpm to 103 bpm, respiratory rate from 18/min to 25/min, and systolic blood pressure from 108 mmHg to 163 mmHg. The patient was awake, alert, and out of bed to a chair. She was less drowsy than previously but remained somewhat slow to respond. She was alert and oriented times two. She was in no acute distress and had no conversational dyspnea. Cardiac examination showed a regular rate and rhythm without murmurs, rubs, or gallops. The lungs were clear to auscultation bilaterally. The abdomen was soft, nontender, and nondistended with normal bowel sounds. The extremities had 2+ distal pulses, no edema, and 2/5 strength in the upper right extremity, 5/5 strength in the upper left extremity, and bilateral lower extremities. She moved all extremities with intact sensation. She reported ongoing back pain following the ALIF.

On hospital day 6, two days after removal of the peripheral catheter, nursing and physician staff identified multiple tense bullae over the right anterior forearm, ranging from a few millimeters to approximately 3 cm in diameter. There was associated swelling and erythema. Several bullae had deroofed, leaving areas of superficial denudation. The lesions were distal to the antecubital catheter insertion site. The patient reported forearm tenderness had been present since hospital day 1 and had progressively worsened. On examination, the right arm was moderately more swollen than the left. Distal pulses were intact. Sensation was intact. She was unable to move the right arm fully. This limitation was due to swelling and a sensation of heaviness rather than acute pain. She endorsed some pain, but only on attempted movement of the right arm. The appearance of the right forearm on hospital day 6 is shown in Figure 1.

**Figure 1:**
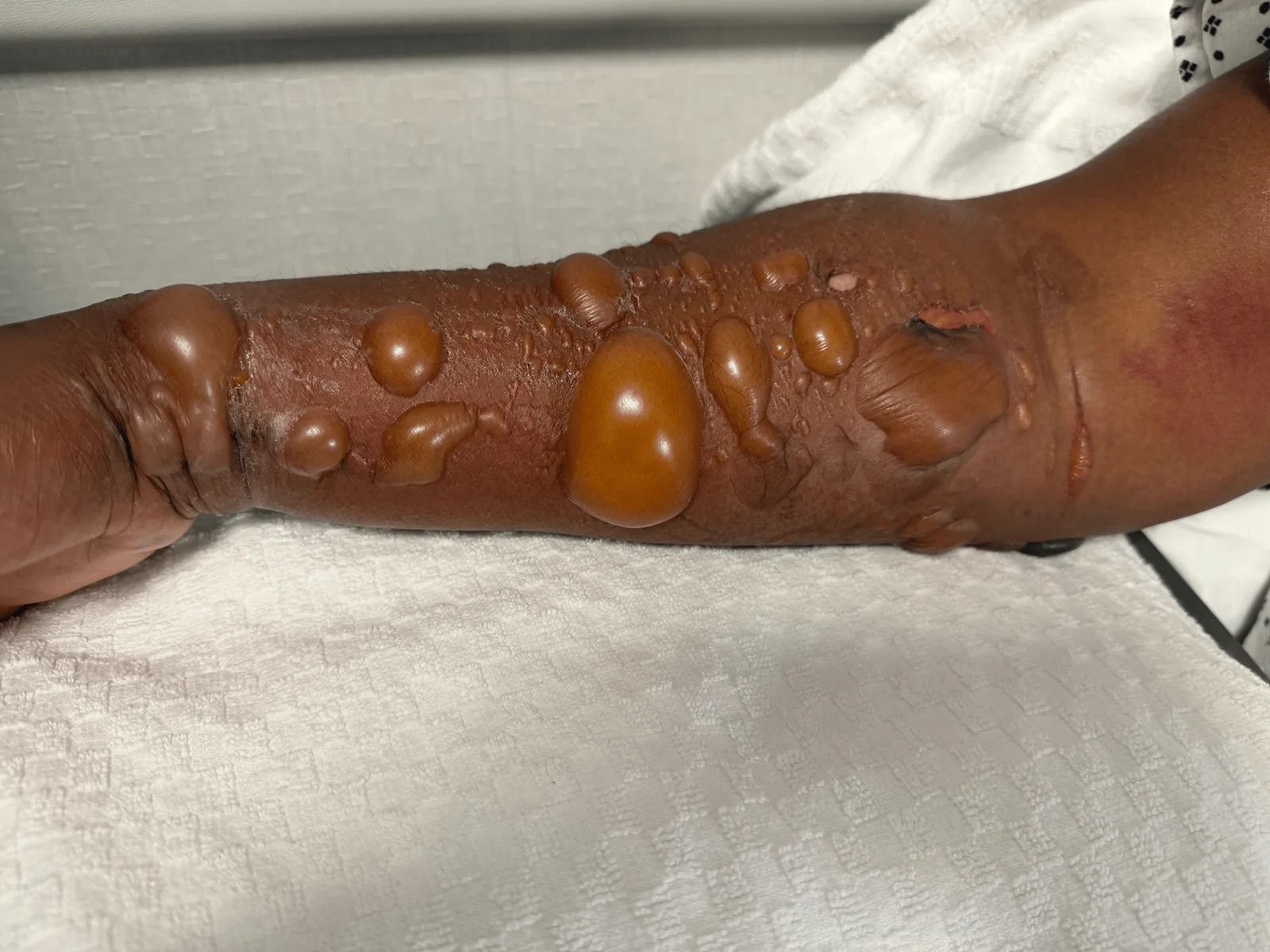
Right anterior forearm on hospital day 6 Multiple tense bullae of varying size are present over the volar forearm, distal to the antecubital catheter insertion site, with intervening areas of superficial epidermal separation. Several bullae had deroofed, leaving areas of denudation with an exposed base. Surrounding skin demonstrates edema and dusky discoloration.

Alternative causes were considered. Right upper extremity venous and arterial ultrasound was normal, excluding deep vein thrombosis and arterial compromise. Compartment pressures were measured and were within normal limits, excluding compartment syndrome. Regarding infection, the patient remained afebrile throughout, with no purulence, fluctuance, lymphangitic streaking, or hemodynamic instability. The leukocytosis was present from admission and downtrended without antibiotic therapy, a pattern more consistent with a postoperative inflammatory response than with an evolving infection. No antibiotics were administered at any point during the admission. The catheter tip was not sent for culture, and blood cultures were not obtained. Based on the temporal relationship to the infiltrated catheter, the anatomic distribution of the lesions, and the exclusion of vascular and infectious causes, the findings were felt to be most consistent with hyperosmolar dextrose extravasation injury. This attribution was clinical rather than confirmed histopathologically.

Management of the extravasation was conservative. Wound care was consulted. Limb elevation was initiated. A compression wrap was applied. Hyaluronidase was not administered. No surgical intervention was performed. At the time of reporting, the patient remained in the ICU because of the every-2-hour capillary glucose monitoring requirement. Localization studies for a suspected insulinoma were ongoing. The extravasation lesions remained present under conservative management. The clinical course, dextrose exposure, and catheter dwell period are summarized in Figure 2.

**Figure 2:**
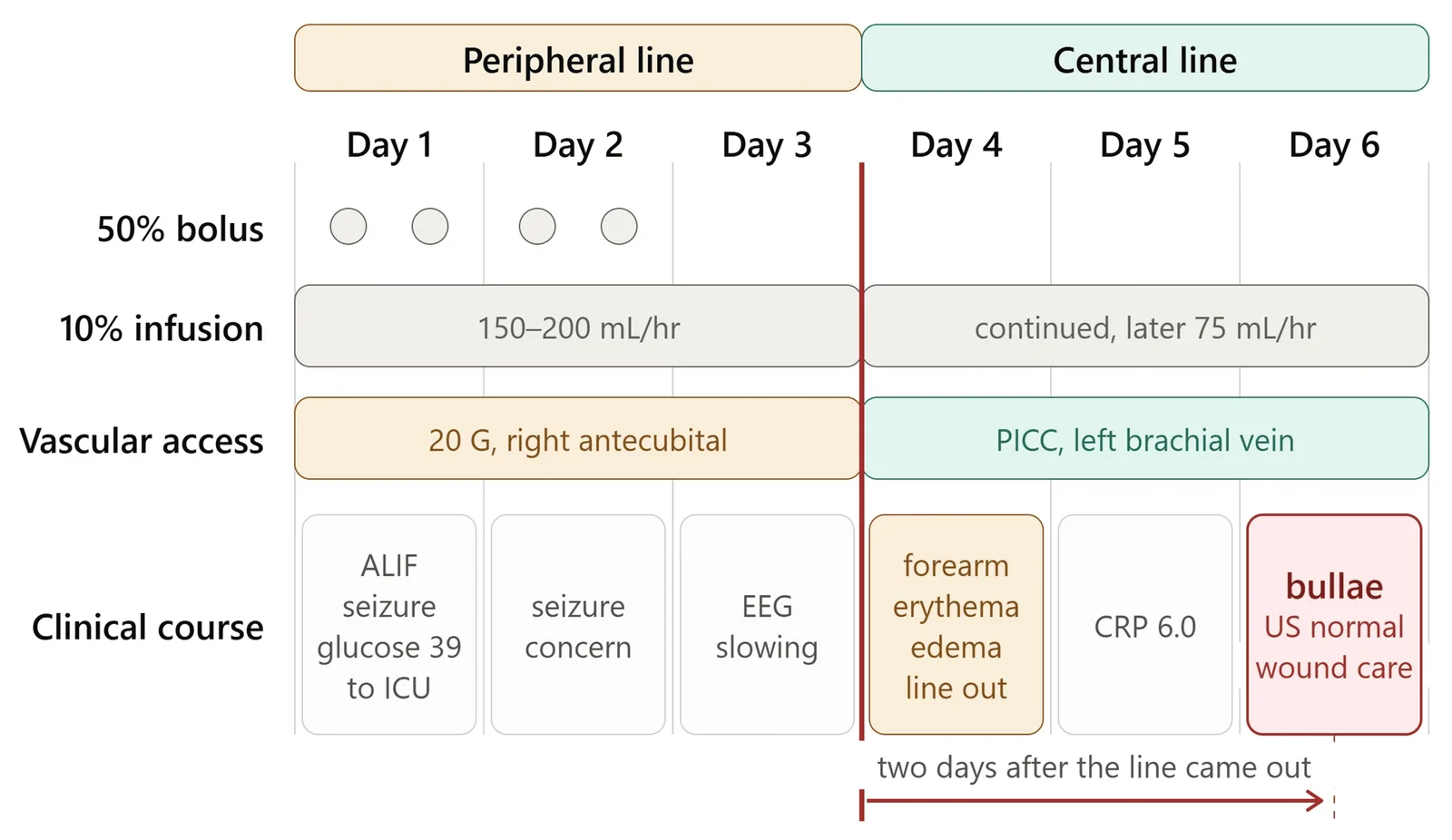
Timeline of the hospital course The continuous 10% dextrose infusion and all four 50% dextrose boluses were administered through a single 20-gauge peripheral catheter in the right antecubital fossa, placed on hospital day 1 and removed on hospital day 4 when the site was found to be infiltrated. A peripherally inserted central catheter was placed in the left brachial vein the same day, and the dextrose infusion was continued centrally. Bullous lesions were identified on hospital day 6, two days after the peripheral catheter had been removed and while the infusion was running centrally. ALIF: anterior lumbar interbody fusion, ICU: intensive care unit, EEG: electroencephalography, PICC: peripherally inserted central catheter, CRP: C-reactive protein, US: ultrasound Created using Microsoft PowerPoint

## Discussion

Convergence of established peripheral extravasation risk factors

Peripheral catheter extravasation is a recognized complication with defined risk factors. Simin et al. reported that a 20-gauge catheter, two or more cannulation attempts, and administration of a high-risk solution on the first day of dwell were each independently associated with infiltration on multivariate analysis [7]. Catheter gauge and dwell duration were independently linked to phlebitis in the same cohort [7]. Marsh et al. pooled data from 76,977 catheters and found an infiltration or extravasation rate of 13.7%, second only to phlebitis among noninfectious complications [6]. This case is notable because more than one of these risk factors was present in a single access site.

Two of the infiltration risk factors identified by Simin et al. were present in this patient. The access was a 20-gauge catheter, and a high-risk hypertonic solution was administered on the first day of dwell, with all four 50% dextrose boluses delivered within the first 48 hours. The catheter remained in place for three days, during which continuous hypertonic infusion ran through the same vein between boluses.

The mechanism links these factors to the observed injury. Shibata et al. showed in a rat model that glucose behaved as an irritant at osmolarities of roughly 833-1,110 mOsm/L and as a vesicant at approximately 1,388 mOsm/L or higher, producing ulceration and necrosis within 24 hours [3]. Lawson et al. reported the osmolality of 50% dextrose at 2,520 mOsm/kg [9]. That value sits well into the vesicant range. Each bolus therefore delivered a vesicant-range solution through a small-bore peripheral vein. Between boluses, the continuous 10% dextrose at 505 mOsm/L maintained a sustained hypertonic load on the same vessel [4]. The severity of noncytotoxic extravasation tracks with osmolarity and duration of tissue contact [13]. The combination here was distinct from either exposure alone. Intermittent vesicant-range boluses were layered onto continuous hypertonic infusion through one small catheter.

The anatomic pattern supported this interpretation. The bullous lesions developed on the anterior forearm, distal to an antecubital insertion site. This distribution is consistent with extravasated fluid tracking distally along the subcutaneous plane. The normal venous and arterial ultrasound excluded deep vein thrombosis and arterial compromise, and compartment pressures were within normal limits. Extravasation therefore remained the most consistent explanation after alternative vascular and infectious causes were considered.

Prolonged infusion versus single bolus as an evidence gap

The published dextrose extravasation literature centers on single boluses. Chinn and Colella described forearm compartment syndrome after a single prehospital 50% dextrose extravasation [8]. Lawson et al. described hyaluronidase treatment after a single 50% dextrose infiltration in the emergency department [9]. Both involved one acute event rather than sustained exposure. This case differed in kind. The exposure spanned days of continuous hypertonic infusion punctuated by repeated vesicant boluses through the same catheter.

Broader literature on prolonged hypertonic dextrose extravasation is sparse. The closest analogue is parenteral nutrition extravasation. The American Society for Parenteral and Enteral Nutrition notes that tissue damage from parenteral nutrition extravasation depends on pH, osmolarity, electrolyte content, and duration of tissue exposure [14]. No dedicated case series describing extravasation from prolonged continuous dextrose infusion for hypoglycemia was identified in this search. That absence itself defines a gap.

Patients with hyperinsulinemic hypoglycemia form a distinct at-risk group. Refractory hypoglycemia from endogenous hyperinsulinism often requires hospitalization with continuous intravenous dextrose while artificial nutrition is established or definitive treatment is arranged [12]. Localization and surgical or tumor-directed therapy take time [11]. The dextrose exposure is therefore measured in days, not minutes. This prolonged bridging requirement is precisely what converts a routine peripheral infusion into a sustained hypertonic insult.

Vascular access selection and prevention

Osmolarity thresholds for access selection are established. The American Society for Parenteral and Enteral Nutrition recommends central venous access for admixtures exceeding 900 mOsm/L and permits peripheral delivery at or below that limit [5,15]. The tension in this case is that no single solution neatly triggered the rule. The continuous 10% dextrose at 505 mOsm/L sits below the threshold [4]. The 50% dextrose boluses sit far above it [9]. Each was delivered peripherally.

The more useful question concerns cumulative hypertonic exposure through one access point over time, not the osmolarity of any single agent. Anticipated duration of therapy warrants weight in access planning. Existing guidance already points this way. The Centers for Disease Control and Prevention recommend a midline catheter or peripherally inserted central catheter rather than a short peripheral catheter when intravenous therapy is expected to exceed six days [16]. For a patient facing a multiday dextrose bridge, early central or midline access is a reasonable preventive step.

Early hyperosmolar extravasation may produce only subtle local change that is difficult to separate from routine infusion site irritation. In this case, the injury became clinically apparent only once bullous lesions formed. The interval between the reported onset and clinical identification reflects the insidious course of this injury rather than any single point of failure. In this case, the bullae appeared two days after the catheter had been removed and the infusion transferred to central access. Tissue injury from hyperosmolar extravasation may therefore continue to evolve after the offending catheter is no longer in use. Structured, scheduled infusion site assessment for patients on prolonged hypertonic therapy is a practical safeguard, alongside early consideration of central access when high-concentration dextrose is expected to continue.

Management of hyperosmolar dextrose extravasation is largely supportive. Standard measures include stopping the infusion, catheter removal, limb elevation, and wound care [14]. Hyaluronidase has been described to aid dispersion and absorption after dextrose and other hyperosmolar extravasations [9,17]. Consensus recommendations from the infusion nursing literature support warm compresses and hyaluronidase for vesicants that injure tissue through an osmolar mechanism [18]. Surgical intervention is reserved for compartment syndrome or established necrosis [8]. In this patient, conservative management with catheter removal, transfer of the infusion to central access, elevation, compression, and wound care consultation was pursued. Hyaluronidase was not administered, and no surgical intervention was required.

Limitations

Several limitations apply. This is a single case, so no causal inference can be drawn, and the attribution of the forearm lesions to dextrose extravasation is a clinical judgment rather than a proven causal diagnosis. Endogenous hyperinsulinism was supported biochemically, but a sulfonylurea screen was not obtained, and pancreatic localization had not been completed at the time of reporting, so insulinoma remained a presumptive diagnosis. The patient was noncompliant with her prescribed antiepileptic therapy, and breakthrough seizure activity related to nonadherence cannot be excluded as a contributor to the seizures independent of hypoglycemia. The catheter tip was not sent for culture, and blood cultures were not obtained. Although the clinical course was not suggestive of infection, obtaining cultures could have provided additional reassurance and more definitively excluded an infectious etiology. No histopathology was obtained, so alternative causes of the soft tissue injury cannot be entirely ruled out. Hyaluronidase was not administered, so this report cannot address its efficacy. Serial photographic documentation of the early lesions was not available, and the precise onset of tissue injury was not objectively documented, relying instead on patient report. The patient was transferred out of the intensive care unit before the lesions resolved, and no follow-up wound care documentation was available, so the long-term outcome and healing course are unknown.

## Conclusions

Prolonged peripheral delivery of high-concentration dextrose was associated with a significant bullous soft tissue injury in this patient. A 20-gauge peripheral catheter carried both continuous 10% dextrose at 505 mOsm/L and four 50% dextrose boluses with an osmolality of 2,520 mOsm/kg over a three-day dwell. Two independently recognized risk factors for peripheral infiltration coincided in one access site. These were the small-bore catheter and delivery of a hypertonic vesicant solution on the first day. The resulting tense bullae developed on the anterior forearm, distal to the antecubital insertion site. Normal venous and arterial ultrasound excluded thrombosis and arterial compromise, and compartment pressures were within normal limits. Extravasation of hyperosmolar dextrose remained the most consistent explanation after alternative vascular and infectious causes were considered, although this attribution was clinical rather than confirmed.

Anticipated duration of hypertonic therapy deserves weight in vascular access planning. The osmolarity of the initial solution alone does not capture the risk. Cumulative hypertonic exposure through a single peripheral vein accrues over days of treatment. Patients who need a multiday dextrose bridge for suspected endogenous hyperinsulinism are a distinct at-risk group. For these patients, early consideration of central or midline access is a reasonable preventive measure derived from this case and from existing vascular access guidance, rather than a recommendation validated specifically for prolonged dextrose therapy. Structured, scheduled infusion site assessment is a practical safeguard during prolonged hypertonic therapy. Early hyperosmolar extravasation can produce only subtle local change, and the injury may become evident only once blistering appears. The optimal vascular access strategy for prolonged dextrose therapy has not been established in comparative studies. The true frequency of this complication in patients receiving a sustained dextrose bridge remains unknown.
